# Enhancing genetic mapping of complex genomes through the design of highly-multiplexed SNP arrays: application to the large and unsequenced genomes of white spruce and black spruce

**DOI:** 10.1186/1471-2164-9-21

**Published:** 2008-01-18

**Authors:** Nathalie Pavy, Betty Pelgas, Stéphanie Beauseigle, Sylvie Blais, France Gagnon, Isabelle Gosselin, Manuel Lamothe, Nathalie Isabel, Jean Bousquet

**Affiliations:** 1 Arborea and Canada Research Chair in Forest and Environmental Genomics, Centre d'Étude de la Forêt, Pavillon Charles-Eugène-Marchand, Université Laval, Québec, Québec G1V 0A6, Canada; 2 Natural Resources Canada, Canadian Forest Service, Laurentian Forestry Centre, 1055 Rue du P.E.P.S., C.P. 10380, succ. Saint-Foy, Québec, Québec G1V 4C7, Canada

## Abstract

**Background:**

To explore the potential value of high-throughput genotyping assays in the analysis of large and complex genomes, we designed two highly multiplexed Illumina bead arrays using the GoldenGate SNP assay for gene mapping in white spruce (*Picea glauca *[Moench] Voss) and black spruce (*Picea mariana *[Mill.] B.S.P.).

**Results:**

Each array included 768 SNPs, identified by resequencing genomic DNA from parents of each mapping population. For white spruce and black spruce, respectively, 69.2% and 77.1% of genotyped SNPs had valid GoldenGate assay scores and segregated in the mapping populations. For each of these successful SNPs, on average, valid genotyping scores were obtained for over 99% of progeny. SNP data were integrated to pre-existing ALFP, ESTP, and SSR markers to construct two individual linkage maps and a composite map for white spruce and black spruce genomes. The white spruce composite map contained 821 markers including 348 gene loci. Also, 835 markers including 328 gene loci were positioned on the black spruce composite map. In total, 215 anchor markers (mostly gene markers) were shared between the two species. Considering lineage divergence at least 10 Myr ago between the two spruces, interspecific comparison of homoeologous linkage groups revealed remarkable synteny and marker colinearity.

**Conclusion:**

The design of customized highly multiplexed Illumina SNP arrays appears as an efficient procedure to enhance the mapping of expressed genes and make linkage maps more informative and powerful in such species with poorly known genomes. This genotyping approach will open new avenues for co-localizing candidate genes and QTLs, partial genome sequencing, and comparative mapping across conifers.

## Background

Single nucleotide polymorphims (SNPs) have become a genomic commodity as they are becoming indispensable in various genome scans aimed at mapping genomes [1-6], finding associations with complex traits [7-10], and population genomics [11,12]. They are distributed along the various regions of the genomes [13,14] and are frequent in coding regions of angiosperms [15,16] and conifers [17-19]. However, the efficiency of genome scans is not only dependant on a wide genomic distribution of SNPs. Indeed, it also relies on the ability to genotype large numbers of SNPs over large sets of individuals.

SNP genome scans in non model species usually involve two steps: the discovery of SNPs and genotyping. With no *a priori *knowledge of DNA polymorphisms, SNPs are usually discovered through various strategies of individual or pool DNA sequencing [20], or by using tilling techniques, a high-throughput strategy relying on the enzymatic cleavage of mismatches [21]. For a number of crop species, current resequencing efforts have led to the development of SNP databases and generate a wealth of SNPs usable in genome scans. In conifers, large-scale EST sequencing projects have been initiated [22-25], providing a starting point to develop SNP resources in pine [17] and spruce [18].

Several SNP genotyping array approaches have been developed with variable success. The accuracy of innovative SNP genotyping technologies has been assessed mostly through the development of assays suitable for analysing variations in the human genome. Broadly speaking, four reaction principles govern SNP genotyping assays: hybridization with allele-specific oligonucleotide probes, oligonucleotide ligation, single nucleotide primer extension, and enzymatic cleavage reviewed in [26-28]. Among these approaches, the GoldenGate assay developed by Illumina and relying on the bead array technology has demonstrated high performance with high levels of call rate, reproducibility, and overall success rate for the analysis of the human genome [29-31].

High-throughput SNP assays have recently been applied to plants. Large datasets of SNP-based markers are being developed in barley through the development of genotyping assays relying on Illumina's technologies [32], leading to the undertaking of an international SNP project [33]. The same genotyping approach has made it possible to map large datasets of SNPs even in complex and duplicated genomes such as soybean [34], and projects are underway in hexaploid wheat [35] and poplar [36].

In the present study, we are asking whether high-throughput SNP genotyping technologies developed for human population genomics applications, such as the Illumina GoldenGate SNP assay, are applicable to large and essentially unsequenced genomes as seen in conifers. Conifer genomes reach very large sizes, around 10,000–40,000 Mb [37], consisting mostly of repetitive sequences [38]. For the two conifers considered herein, white spruce and black spruce, genome sizes are well in excess of 10^e^10 bp [39].

Moreover, the partial knowledge of the large and redundant genomes of conifers can be a limiting factor to design an efficient SNP genotyping assay. Indeed, sequences located upstream and downstream the SNP cannot be fully validated for locus specificity and the possible presence of repetitive elements [29,30]. The possible effect of such a drawback remains to be verified for most crop and tree species which genomes are essentially not sequenced. Based on EST sequence data available for white spruce [40], we have designed primers and resequenced genomic DNA for hundreds of genes in white spruce and black spruce. The high quality SNP datasets developed were used to select SNPs amenable to the GoldenGate genotyping assay and test the technology for these two species. Then, we integrated these SNP data into linkage maps of expressed genes and illustrated the possibility to rapidly improve the density of existing genetic maps for spruce species.

## Methods

### Plant material for genotyping assays and linkage mapping

Plant material consisted of one outbred F_1 _cross # C9612856 (♀80112 × ♂80109) for white spruce (*Picea glauca *[Moench] Voss) with 292 progeny derived from two parents selected for their high level of heterozygosity for ESTP anchor markers and for their intermediate performance for a number of traits such as somatic embryogenic capacity [41]. Plant material for black spruce (*Picea mariana *[Mill.] B.S.P.) was representative of the hybridizing species complex *Picea mariana × Picea rubens *(hereafter designated as black spruce) and consisted of 283 progeny derived from the backcross-like cross BC1 (#9920002: ♀11307-03 [♀83 × ♂425] × ♂425), as previously described [42]. Spruces all harbor 2n = 2x = 24 chromosomes, as for most Pinaceae [43,44]. The lineages leading to these two taxa are thought to have diverged at least 10 Myr ago [45].

### Gene selection and PCR amplification

For SNP discovery, we considered a collection of 16,500 unigenes derived from a white spruce EST database [40]. For each gene, coding regions were identified based on alignments with similar sequences from uniprot-swissprot protein databases. PCR primers for amplification and resequencing were designed using Primer3 [46]. Whenever possible, one of the primers was anchored outside of the coding regions in order to increase amplification specificity. The same primers were also used for SNP discovery in black spruce. The lists of unigene and primer sequences for the genes represented on the two Illumina SNP bead arrays for white spruce and black spruce are provided [see Additional file 1].

For each of white spruce and black spruce, DNA was isolated from the two parents of the mapping population and their progeny, as well as from haploid megagametophyte tissue using a DNeasy Plant Mini Kit (Qiagen, Mississauga, Ontario). About 5–20 ng of template DNA were used for PCR amplification. Reactions were done in 30 μl containing 20 mM Tris-HCl (pH 8.4), 50 mM KCl, 1.5–2.5 mM MgCl_2_, 250 μM of each dNTP, 400 μM of both 5' and 3' primers and 1.0 unit of Platinum *Taq *DNA Polymerase (Invitrogen). Peltier Thermal Cycler (DNA Engine, DYAD™, MJResearch) was used with a initial denaturation of 4 minutes at 94°, followed by 35 cycles of 30 seconds at 94°, 30 seconds at annealing temperature optimized between 55 and 60° for each pair of primers, and 3 minutes at 72°, completed with an additional 10 minutes at 72°.

Each PCR fragment was sequenced with the amplification primers using BigDye Terminator v3.0 cycle sequencing ready reaction kit (Applied Biosystems, Foster City, California) and an automated ABI Prism^® ^3700 Genetic Sequencer (Applied Biosystems). Sequences were analysed and assembled with Seqmerge (Genetics Computer Group, Wisconsin Package Version 10.3, Accelrys, San Diego, California).

### SNP discovery

SNPs were detected for each of the two parents for each species from heterozygous positions indicated by double-peak signatures in sequence chromatograms. For each SNP detected, haploid DNA sequences from individual megagametophyte tissue were used as a control for paralogy. Because of their haploid nature, any double peak signature in the sequence chromatogram from a megagametophyte would indicate a SNP resulting from polymorphism between gene loci, hence paralogous variation. Because these SNPs would result in fixed polymorphisms in the progeny, they were not considered for genotyping and mapping.

### SNP selection for bead array construction

Gene regions were amplified by PCR and resequenced in order to identify in excess of 1,000 candidate SNPs for each of white spruce and black spruce. Out of them, 1,534 SNPs were used to construct two species-specific Illumina bead arrays of 768 SNPs using the GoldenGate assay (Illumina Inc., San Diego, California). For each species, when more than one SNP was available for a gene, SNPs were chosen so each SNP was specific to one parental genotype. This scheme allowed to validate the SNP mapping approach, as SNPs of a same gene are expected to map at the same position on composite maps. In optimizing the choice of SNPs for a given gene in a given species, those with maximum GoldenGate assay functionality score were chosen. The functionality score is an *a priori *measure of SNP adequation to the GoldenGate assay and takes into account a number of parameters, including sequence conformation around the SNP, lack of repetitive elements in the surrounding sequence (200 bp upstream and downstream) and sequence redundancy against the available sequence database of the recipient species [29]. For white spruce, a small subset of 38 SNPs representative of 31 genes was drawn from *in silico *identification of SNPs in contigs resulting from the assembly of EST sequences [18]. All chosen *in silico *SNPs involved EST sequences from at least two different cDNA clones and had a probability of occurrence of 0.95 or more, according to the statistical assessment conducted with a bayesian method [18].

### SNP genotyping assay

The Illumina bead array technology was used to carry out all genotyping reactions in accordance with the manufacturer's protocol for the SNP GoldenGate assay [29]. Highly multiplexed allele-specific extension reactions were conducted with two allele-specific primers per SNP for each of the two species-specific 768-SNP arrays using 250 ng of template DNA per sample (at a rate of 50 ng/μl) for each progeny in each species and for positive controls consisting of five replicates of each parent of the mapping populations also used to identify SNPs from resequencing. Negative controls were also added to each 96-well sample plate. Ligation was completed with a third locus-specific primer. This step was followed by PCR amplification on the extension-ligation product using primers labeled with either Cy3 or Cy5 dye to distinguish between alleles at each SNP. Products of the PCR reaction were hybridized onto a decoded Sentrix Array Matrix (SAM) (Illumina Inc., San Diego). Bundles of the SAM include beadtypes coated with oligonucleotides complementary to a primer address on the PCR product. Each beadtype is represented with an average redundancy of 30X on the array to optimize the accuracy of the final genotype signal. Following hybridization, the signal in each wavelength was determined using a bead array reader that converts images to intensity data. The intensity data for each SNP was normalized and assigned a cluster position (and resulting genotype) with the BeadStudio software (Illumina Inc.), and a quality score for each genotype was generated. A GenCall score cutoff of 0.25 was used to determine valid genotypes at each SNP and the SNPs retained had to get a minimum GenTrain score of 0.25 [47,30]. Gentrain scores measure the reliability of SNP detection based on the distribution of genotypic classes [30]. DNA reports, locus summaries, and final reports were generated for downstream analysis using the BeadStudio software (Illumina Inc.).

### Estimation of linkage maps

AFLP, RAPD, SSR, and ESTP marker data previously used for linkage mapping in the two species [20,41,42] were considered together with the new SNP data for constructing linkage maps. For each cross, locus segregation was tested for goodness-of-fit to expected Mendelian segregation ratios using chi-square tests (P ≤ 0.01 with Bonferonni correction). Distorted loci were excluded from further analyses. Linkage analyses were conducted with the male and female datasets independently to obtain two individual linkage maps for each species. Each SNP was considered as an independent marker. Then, a composite linkage map was assembled from the two parental maps for each species, where SNPs from the same gene were considered simultaneously as a single haplotype. Individual and composite linkage maps were estimated using procedures described by Pelgas et al. [42]. Both crosses were analysed using the "two-way pseudo-testcross" mapping approach [48]. All linkage analyses and map estimations were performed with JoinMap 3.0 [49,50]. In addition, markers were ordered with the Monte Carlo maximum likelihood mapping algorithm implemented in JoinMap 4.0 and using standard parameters [51]. With both versions of JoinMap, the parameter CP (cross-pollination) was used with a maximal threshold value of 5 for the jump, a ripple value of 1, and Kosambi's mapping function [52]. For marker grouping and linked loci ordering, a LOD score of 6.0 to 9.0 and a minimum recombination fraction (*θ*) of 0.30 were used. The expected genome map length *Ge *was estimated under the assumption of random marker distribution according to the formula of [53]. An estimate of genome map coverage *Ce *was obtained according to the formula of [54] for the same LOD value (4.0, used for individual linkage maps) as for previous coverage estimates [41,42].

### Distribution of markers on linkage groups

For each species, randomness of gene locus distribution within and among linkage groups, heterogeneity of marker distribution (G-tests) among linkage groups, and marker dispersion were analysed from the composite map as previously described [46]. Analyses were conducted by considering 1) all marker types and 2) only gene markers (SNPs and ESTPs). For AFLP markers only, previously published analyses of randomness of distribution showed no aggregated pattern of distribution [46,41]. As no additional AFLP markers were added in the present study, no distribution test was conducted with this type of marker alone.

### Validation of marker orthology between species

The homoeology of linkage groups between white spruce and black spruce was determined according to the same criterion as described by Pelgas et al. [41]. The recognition of orthologous from paralogous loci was also carried out according to Pelgas et al. [41]. To validate exceptions to linkage group synteny, resequencing from haploid megagametophyte tissue was performed for presumed orthologous markers positioned on non-homoeologous linkage groups. Any sequence polymorphism detected in the chromatogram of the haploid DNA sequence was considered as evidence for paralogy. Changes in synteny were validated on a second mapping population available for each species whenever necessary.

## Results

### Construction of SNP-arrays

A total of 487 expressed sequences were amplified and resequenced in white spruce parents, of which 394 were found with at least one orthologous SNP (Table 1). Primers designed for amplification and resequencing of expressed genes in white spruce could be transferred to black spruce at a rate of 90.1%, which is in line with results obtained previously for a more limited set of genes [55]. The transfer procedure resulted in the amplification and sequencing of 462 genes in black spruce parents, of which 355 contained at least one orthologous SNP (Table 1). For the 279 genes simultaneously amplified, resequenced, and found with at least one SNP in each of white spruce and black spruce, only 4.7% of the observed SNPs were shared between the two species (Table 1). This fraction is based on gene resequencing from two parents in each species. Accordingly, two Illumina bead arrays of 768 SNPs for the GoldenGate assay were constructed, one for each species. The array built for white spruce contained SNPs representative of 425 genes and that for black spruce contained SNPs for 348 genes. The Additional file 1 (Table S1) provides primer sequences used for PCR amplification, unigene identifiers, and links with the ForestTreeDB database hosting the unigene sequences and their annotations [56]. A total of 273 genes were represented simultaneously on both arrays [Additional file 1]. All in all, the white spruce SNP-array resulted in 232,000 SNP calls and the black spruce SNP-array in 225,000 SNP calls.

**Table 1:** Sequence production for the SNP discovery step. Sequence production for the SNP discovery step for each of white spruce and black spruce.

Production parameter	White spruce	Black spruce	In common between the two species
Number of genes successfully amplified and resequenced	487	462	457
Number of genes with orthologous SNPs	394	355	279
Total number of orthologous SNPs	1102	959	45
Number of genes on species-specific SNP array	425	348	273
Number of resequenced SNPs on species-specific SNP array	730	768	14
Number of *in silico *SNPs^1 ^on species-specific SNP array	38	-	-

^1 ^*in silico *SNPs were detected in aligned ESTs derived from white spruce cDNA libraries. The resource is described in Pavy et al. [48]. For black spruce, all assayed SNPs were obtained after resequencing from genomic DNA.

### Reproducibility of the SNP assay and effect of template concentration

The reproducibility of the assay was evaluated using five replicates for each of the mapping parents also used to identify SNPs from resequencing. When estimated over all valid SNPs (thus excluding failed SNPs, see below), on average, 99.4% of the SNP calls were concordant with expectations when using the recommended amount of DNA template (50 ng/μl in 5 μl). The rate of concordant SNP calls was lower when testing with less template DNA (97.8% for 17 ng/μl, 94.3% for 10 ng/μl, and 81.2% for 4 ng/μl, all assays in 5 μl sample volume).

### Overall success rate of the SNP bead arrays

GenTrain scores correspond to the reliability of SNP detection based on the distribution of genotypic classes. Thus, it is a measure of reliability based on the total array of calls for a given SNP. According to Illumina, for a SNP to be retained, a minimum GenTrain score of 0.25 is advisable [30,47,57]. In the present study, a SNP had to get a minimum GenTrain score of 0.25 and had to be segregating in the related mapping population to be declared successful. In white spruce, 81.6% of SNPs identified by resequencing had GenTrain score of 0.25 or more (Table 2), which is in the range of that obtained for human SNPs identified from resequencing for polymorphism discovery [29]. In black spruce, the corresponding percentage was 82.0%. Contrary to expectations, a number of these SNPs were monomorphic in the mapping populations (Table 2). It is likely that one of the two allele-specific primers in the GoldenGate assay defaulted for these SNPs. When discarding these monomorphic SNPs, the overall rate of success for the genotyping of resequenced SNPs was 69.2% in white spruce and 77.1% in black spruce (Table 2). The genotyping success rate on the basis of the number of genes assayed was higher (respectively 77.6% and 89.4% for each of white spruce and black spruce, Table 2) because of redundancy of SNP sampling for some genes.

**Table 2:** Success rate. Success rate obtained over 768 SNPs assayed for each of white spruce and black spruce using the GoldenGate SNP assay. Numbers in parentheses are the percentages obtained by using as a reference the total of 768 SNPs assayed per species or the total number of genes assayed.

		On a SNP basis				On a gene basis	
		Number of SNPs assayed	Number of SNPs with GenTrain score ≥ 0.25 ^2^	Number of segregating SNPs with GenTrain score ≥ 0.25 ^2^	Number of monomorphic SNPs with GenTrain score ≥ 0.25 ^2^	Number of genes assayed	Number of segregating genes

White spruce							
	Resequenced SNPs	730 (95%)	596 (81.6%)	505 (69.2%)	62 (8.5%)		
	*in silico *SNPs ^1^	38 (5%)	31 (81.6%)	11 (28.9%)	25 (65.8%)		
	Total	768 (100%)	603 (78.5%)	516 (67.2%)	87 (11.3%)	425	330 (77.6%)
Black spruce							
	Resequenced SNPs	768 (100%)	630 (82.0%)	592 (77.1%)	31 (4.0%)	348	311 (89.4%)

^1 ^*in silico *SNPs were detected in aligned EST derived from white spruce cDNA libraries. The resource is described in Pavy et al. [18]. For black spruce, all SNPs were obtained after resequencing from genomic DNA.

^2 ^For SNPs with a GenTrain score ≥ 0.25, valid GenCall scores were obtained for 99.4% of samples, on average (see Results).

For white spruce, a number of *in silico *SNPs identified from redundancy in EST contigs [18] were also included on the SNP array, and 81.6% of them had GenTrain score of 0.25 or more (Table 2), which is comparable to the percentage obtained for resequenced SNPs. The overall success rate taking into account segregation in the mapping population was lower at 28.9%, because monomorphism for *in silico *SNPs was much more frequent than that for resequenced SNPs. Individuals previously used for EST sequencing and *in silico *identification of SNPs did not include the parents of the present white spruce mapping population. Hence, it is likely that white spruce parents were homozygous for many of these *in silico *SNPs, which resulted in a much higher rate of monomorphism than for resequenced SNPs. Indeed, when scoring these *in silico *SNPs for individuals from natural populations, many of these SNPs exhibited the expected polymorphism and the overall success rate obtained was 60% (data not shown).

### Rate of missing data according to SNP GenTrain scores

A missing data is generated when the GenCall score for a particular individual and SNP is below 0.25 [30]. GenCall is a measure of the reliability of an individual SNP call relative to the distribution of genotypic classes for that SNP. The call rate, which is 1 minus the rate of missing data, could be estimated for all SNPs with acceptable GenTrain scores. In agreement with data on human SNPs [30], our results indicated that SNPs with GenTrain scores of 0.25 or more were highly reliable with a low rate of missing data (Table 3). The average call rate per valid SNP with GenTrain score of 0.25 or more was 99.4% for white spruce and 99.6% for black spruce. For SNPs with GenTrain scores higher than 0.4, the rate of missing data became infinitesimal and the average rate of missing data per successful SNP was very low, with an average of 0.61% for white spruce and 0.40% for black spruce (Table 3), thus less than 1%.

**Table 3:** Missing data. Rate of missing data per valid segregating SNP according to classes of GenTrain scores for each of white spruce and black spruce.

Species	Class of GenTrain scores	Number of SNPs assayed	Number of segregating SNPs	Number of monomorphic SNPs	Average number of missing data per SNP scored	Average call rate per SNP scored (%)^1^
White spruce						
	<0.25	165	0	0	-	-
	0.25–0.3	0	0	0	-	-
	0.3–0.4	3	3	0	11.0	96.2
	0.4–0.5	15	10	5	2.2	99.2
	0.5–0.6	46	33	13	1.9	99.3
	0.6–0.7	92	81	11	1.4	99.5
	0.7–0.8	323	302	21	1.6	99.4
	0.8–0.9	118	82	36	2.4	99.1
	>0.90	6	5	1	2.8	100
	Total	768	516	87	-	-
	Weighted average	-	-	-	1.8	99.4
Black spruce						
	<0.25	138	0	0	-	-
	0.25–0.3	0	0	0	-	-
	0.3–0.4	10	10	0	3.5	98.8
	0.4–0.5	17	16	1	4.5	98.4
	0.5–0.6	80	74	6	1.9	99.3
	0.6–0.7	79	72	6	2.3	99.3
	0.7–0.8	170	163	7	0.7	99.8
	0.8–0.9	260	244	16	0.5	99.8
	>0.90	14	13	1	2.0	99.3
	Total	768	592	31	-	-
	Weighted average	-	-	-	1.1	99.6

^1 ^Average call rate is 100% minus average number of missing data in %.

### SNP success rate according to *a priori *SNP functionality score

Before construction of the SNP bead array, a functionality score was calculated for each candidate SNP using the Illumina OligoDesigner software [29]. The functionality score relies much on the uniqueness and lack of repetitive elements in the surrounding sequence of each SNP [29]. The higher the score, the more likely the SNP will be functional at the genotyping stage under the GoldenGate assay. SNPs with a predicted functionality score above 0.60 had a much higher rate of success than those below 0.60 (χ^2 ^= 51.0 with the white spruce SNP data and χ^2 ^= 34.9 with the black spruce SNP data, d.f. = 1, P ≤ 0.01) (Table 4). Thus, in spite of the incompleteness of the spruce genome sequence used to estimate the appropriateness of candidate SNPs for the GoldenGate assay, the functionality score was still a valuable predictor of the likelihood of success of the designed oligonucleotides. Indeed, most of the SNPs selected for arraying had functionality scores equal or higher than 0.60 (Table 4).

**Table 4:** Genotyping success rate. Genotyping success rate according to *a priori *Illumina functionality scores of SNPs for the GoldenGate assay for each of white spruce and black spruce.

Species	Class of functionality scores	Predicted functionality^1^	Number of SNPs on SNP bead array	Number of valid SNPs detected^2^	Overall success rate (%)^2^	Number of monomorphic SNPs	Number of failed SNPs
White spruce							
	0.1–0.4	low	18	8	44.4	2	8
	0.4–0.6	medium	71	27	38.0	6	38
	0.6–1.0	high	679	481	70.8	74	124
	Total	-	768	516	-	82	170
	Weighted average	-	-	-	67.2	-	-
Black spruce							
	0.1–0.4	low	4	2	50.0	0	2
	0.4–0.6	medium	70	37	52.9	3	30
	0.6–1.0	high	694	553	79.7	34	107
	Total	-	768	592	-	37	139
	Weighted averaged	-	-	-	77.1	-	-

^1^According to Illumina OligoDesigner software [29]

^2^Excluding failed and monomorphic SNPs.

### Individual and composite linkage maps for white spruce and black spruce

Depending on the cross and parent analysed, between 518 and 586 genetic markers were available to estimate each individual linkage map (Table 5). Of these, between 86.3% and 91% could be mapped. They were distributed over 12 major linkage groups, except for parent 80109, for which one additional minor linkage group derived exclusively from AFLPs was obtained.

**Table 5:** Parameters of individual and composite linkage maps of white spruce and black spruce.

Mapping parameters	Cross/Parents for white spruce (*Picea glauca*)			Cross/Parents for black spruce (species complex *Picea mariana *× *P. rubens*)		
	Parents		Composite	Parents		Composite

	♀80112	♂80109		♀11307-03 [♀83 × ♂425]	♂425	

Total number of available markers	525	597	1039	587	563	1260
Number of distorted markers^b^	7	11	17	12	3	28
Total number of markers without segregation distortion	518	586	1022	575	560	1232
Total number of assigned markers	509	581	957	534	542	1064
Number of AFLP loci	256	299	581	247	242	679
Number of RAPD loci	0	0	0	2	1	3
Number of SSR loci	9	10	13	27	27	45
Number of ESTP gene loci	22	23	35	26	30	34
Number of SNP gene loci	222	249	328	232	242	303
Number of positioned markers (%)	483 (91.0)	523 (90.0)	821 (85.8)	461 (86.3)	479 (88.4)	835 (78.5)
Number of AFLP loci	242	257	461	185	188	469
Number of RAPD loci	0	0	0	2	1	2
Number of SSR loci	8	9	12	25	27	36
Number of ESTP gene loci	19	19	31	23	27	30
Number of SNP gene loci	214	238	317	226	236	298
Number of positioned accessory markers	4	3	9	0	6	22
Number of major linkage groups (nb of sub-groups) (n > 10 markers)	12 (4^c^)	12 (1^c^)	12	12 (2^c^)	12 (2^c^)	12
Number of minor linkage groups (3 ≤ n ≤ 10 markers)	0	1	0	0	0	0
Number of unlinked markers	9	5	65	41	18	168
Total observed map length *G*_F_, cM (Kosambi)	2146.1	2283.6	2304.2	1833.5	1814.1	1849.8
Average map density, cM (Kosambi)	4.4	4.4	2.8	4.0	3.8	2.2
Average size for major linkage groups, cM (Kosambi)	134.1	163.1	192.1	130.9	129.5	154.1
Expected map length *Ge*, cM (Kosambi)	3204.5	3569.9	-^d^	4009.4	3424.7	-^d^
Map coverage *Ce *(%)	98.3	98.4	-^d^	97.7	98.1	-^d^

^a^For individual linkage maps, only markers segregating 1:1 or 1:1:1:1 were used.

^b^Bonferroni correction: *P *≤ 0.01/number of loci.

^c^Number of linkage group composed of 2 sub-groups having more than 10 markers.

^d^Could not be calculated due to the merging of data.

**Figure 1:**
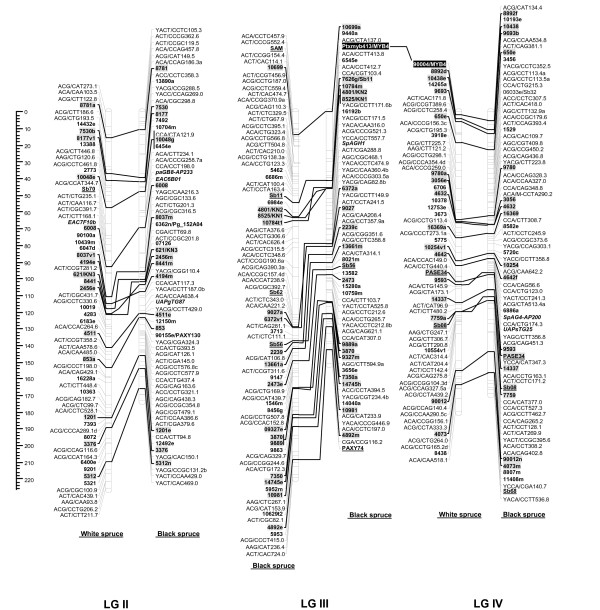
**Comparison of homoeologous linkage groups (LGs) of composite maps for white spruce (on the *left*) and black spruce (species complex *Picea mariana *× *P. rubens*) (on the *right*)**. For each taxon, the composite map was obtained by assembly of two parental datasets and use of JoinMap 3.0 and 4.0. [49,50]. Genetic distances are indicated on the left of the figure (Kosambi). Markers in *bold *are gene SNPs, markers in *bold *and *underlined *are ESTPs, markers in *bold *and *italics *are SSRs, markers in *italic *and *underlined *are RAPDs and all other markers are AFLPs. Markers with a *grey background *are common between both taxa. Orthologous and paralogous markers are connected by a *solid line *and *dashed line*, respectively. Markers not positioned onto homoeologous LGs are printed in white on a black background.

**Figure 2:**
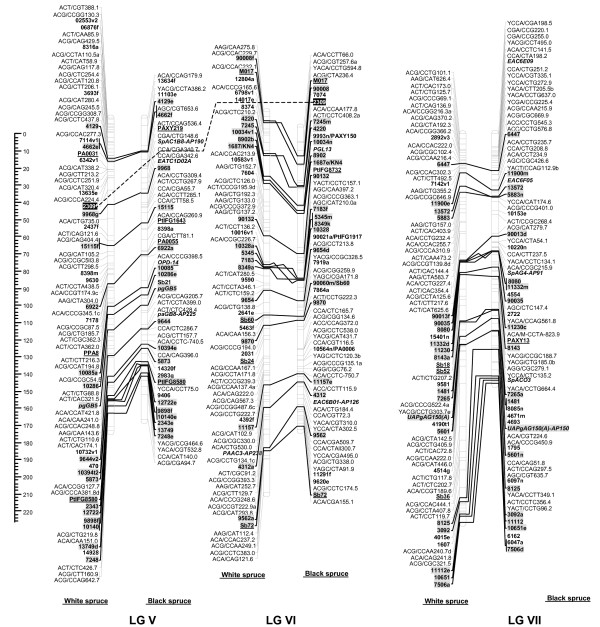
**Comparison of homoeologous linkage groups (LGs) of composite maps for white spruce (on the *left*) and black spruce (species complex *Picea mariana *× *P. rubens*) (on the *right*)**. For each taxon, the composite map was obtained by assembly of two parental datasets and use of JoinMap 3.0 and 4.0. [49,50]. Genetic distances are indicated on the left of the figure (Kosambi). Markers in *bold *are gene SNPs, markers in *bold *and *underlined *are ESTPs, markers in *bold *and *italics *are SSRs, markers in *italic *and *underlined *are RAPDs and all other markers are AFLPs. Markers with a *grey background *are common between both taxa. Orthologous and paralogous markers are connected by a *solid line *and *dashed line*, respectively. Markers not positioned onto homoeologous LGs are printed in white on a black background.

**Figure 3:**
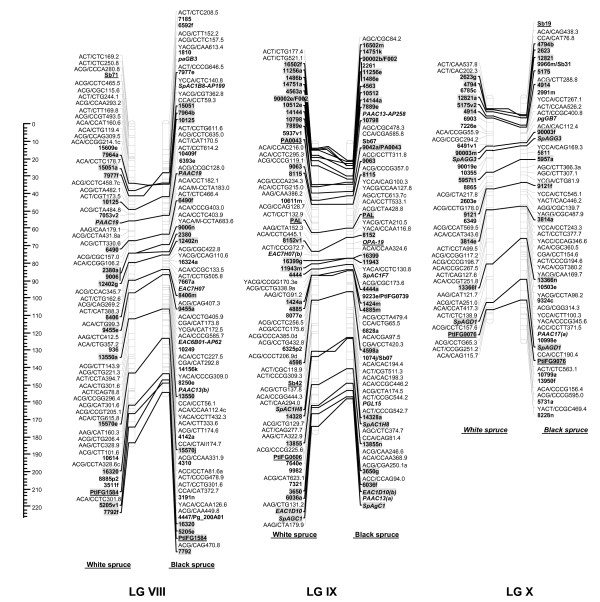
**Comparison of homoeologous linkage groups (LGs) of composite maps for white spruce (on the *left*) and black spruce (species complex *Picea mariana *× *P. rubens*) (on the *right*)**. For each taxon, the composite map was obtained by assembly of two parental datasets and use of JoinMap 3.0 and 4.0. [49,50]. Genetic distances are indicated on the left of the figure (Kosambi). Markers in *bold *are gene SNPs, markers in *bold *and *underlined *are ESTPs, markers in *bold *and *italics *are SSRs, markers in *italic *and *underlined *are RAPDs and all other markers are AFLPs. Markers with a *grey background *are common between both taxa. Orthologous and paralogous markers are connected by a *solid line *and *dashed line*, respectively. Markers not positioned onto homoeologous LGs are printed in white on a black background.

**Figure 4:**
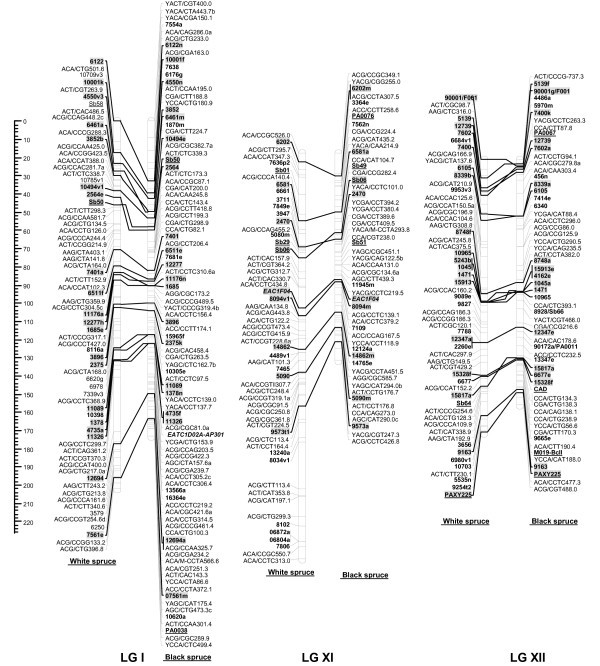
**Comparison of homoeologous linkage groups (LGs) of composite maps for white spruce (on the *left*) and black spruce (species complex *Picea mariana *× *P. rubens*) (on the *right*)**. For each taxon, the composite map was obtained by assembly of two parental datasets and use of JoinMap 3.0 and 4.0. [49,50]. Genetic distances are indicated on the left of the figure (Kosambi). Markers in *bold *are gene SNPs, markers in *bold *and *underlined *are ESTPs, markers in *bold *and *italics *are SSRs, markers in *italic *and *underlined *are RAPDs and all other markers are AFLPs. Markers with a *grey background *are common between both taxa. Orthologous and paralogous markers are connected by a *solid line *and *dashed line*, respectively. Markers not positioned onto homoeologous LGs are printed in white on a black background.

For each cross, male and female datasets were integrated into one composite linkage map representative of each species. For white spruce, 821 markers (461 AFLPs, 12 SSRs, 348 gene markers including 31 ESTPs and 317 SNPs) could be mapped over the 2,304.2 cM, including nine accessory marker loci (Table 5, Figures 1, 2, 3, 4). On this map, the average marker spacing was 2.8 cM. For black spruce, a total of 835 markers (469 AFLPs, 2 RAPDs, 36 SSRs, 328 gene markers including 30 ESTPs and 298 SNPs) could be mapped over the 1,849.8 cM, including 22 accessory marker loci (Table 5, Figures 1, 2, 3, 4). The average marker spacing was 2.2 cM.

### Marker distribution

To analyse the distribution of marker loci, G-tests for goodness-of-fit were used. For each composite linkage map, markers were homogeneously distributed across linkage groups (data not shown). Therefore, coefficients of dispersion could be estimated for each composite map see [42]. Whether we considered all markers or gene loci only (SNPs and ESTPs), the coefficients of dispersion calculated with a sliding window of 3 cM ranged between 0.9 and 1.1 for the white spruce composite map and between 1.1 and 1.3 for the black spruce composite map. Such values around 1.0 indicates the absence of significant marker islands and a random distribution of gene loci at the present mapping intensity.

### Interspecific comparisons

The composite linkage maps of white spruce and black spruce shared 215 homologous anchor markers (9 SSRs, 13 ESTPs, and 193 SNPs). Over the 12 homoeologous linkage groups, 98.1% of homologous markers were in synteny (Figures 1, 2, 3, 4). One ESTP locus (*Ptxmyb413 *= *90004/MYB4*) and three gene SNPs (*2309*, *3656*, and *6047*) were involved in synteny discrepancies. The differential positioning of the ESTP locus was already pointed out and validated by sequencing [41]. The examination of chromatograms obtained from sequencing haploid megagametophyte DNA around the two SNP markers *6047 *and *3656 *respectively positioned onto LGs II/VII and LGs XII/III of white spruce/black spruce revealed polymorphisms. Such evidence indicates that these two anchor markers differentially positioned in white spruce and black spruce correspond to two paralogous gene loci. Another discrepancy involved SNP *2309 *positioned onto white spruce LG V and on black spruce LG VI. However, linkage mapping analyses independently conducted in a second white spruce mapping population (data not shown) led to localize this gene marker on LG VI in both species. Therefore, exceptions to synteny were rejected regarding the differential positioning of these three SNP gene loci between the two species. Along with synteny, macrocolinearity was also well conserved among homoeologous linkage groups between the two spruce taxa. On average, 82% of syntenic anchor markers were positioned in the same order (Figures 1, 2, 3, 4). Minor inversions involving closely spaced markers were observed within each homoeologous linkage group, involving a total of 1 SSR, 6 ESTPs and 31 SNPs. These inversions were also detected when comparing individual linkage maps within species (data not shown), thus lending support to statistical artifacts related to joining individual linkage maps when constructing composite maps.

## Discussion

### Applicability of the GoldenGate SNP assay

The Illumina GoldenGate SNP assay, together with the bead array technology, has been extensively used in studies of human polymorphisms [13]. Its use has also been extended to animal genetics, especially with regard to efforts undertaken by the Bovine HapMap consortium [58]. Because of its well-established reliability with human data and high level of multiplexing, there is a growing interest in using the GoldenGate SNP assay in plants when large numbers of SNPs need to be surveyed. Indeed, the flexibility of its design and the large number of SNPs screened per assay make the technology appropriate for genome scan applications or plant molecular breeding purposes. However, with the exception of reports from biotech companies, very few studies with detailed supportive evidence have been published on the ease of implementing the technology in plants and non model organisms with largely unsequenced genomes. We have considered this issue in spruce by examining the genotyping success rates obtained over a set of high quality SNPs derived from independent resequencing.

#### Call rate

The call rate is the fraction of genotypes called over the possible SNP calls after having excluded unsuccessful assays [30]. In our data, the SNPs retained for genetic mapping analysis had call rates greater than 96%. Their average call rate was in excess of 99%. This value is comparable to call rates obtained with human SNPs using the GoldenGate assay, which are near 100% when following the same stringent criterion of minimum GenTrain score of 0.25 as that used in the present study for considering as valid the genotyping of a SNP [29,30,59,60].

#### SNP conversion rate

The rate of SNPs successfully genotyped using the GoldenGate assay, or SNP conversion rate, is calculated by counting only valid SNPs displaying GenTrain score above a given cutoff. The GenTrain score of a SNP reflects the degree of separation between homozygote and heterozygote clusters and the ease of placement of individual calls within a cluster, which are key measures of signal-to-noise in the assay data [30]. As recommended by Illumina, we followed a conservative approach and filtered out SNPs with a GenTrain score below 0.25, which tend to show low call rate and hence, high rates of missing data [30]. Based on this criterion alone and not considering monomorphic SNPs, the SNP conversion rates were 81.6% and 82.0% of the resequenced SNPs for the white spruce and black spruce SNP arrays, respectively. These rates were down to 69.2% and 77.1%, respectively, when excluding monomorphic SNPs. High SNP conversion rates have been obtained in studies conducted in species with completely sequenced genomes such as for the human genome. Besides studies involving human SNPs, GenTrain score cutoffs have been barely mentioned in the literature although they affect the SNP conversion rates and the average call rate. Thus, the following comparisons of our results with the published literature may not be orthogonal.

In a study encompassing 1,536 resequenced SNPs derived from the human genome, 93.3% of SNPs were called after removal of low frequency SNPs [61]. Two other sets each encompassing 768 HapMap SNPs were successfully assayed with 91.0% and 93.9% conversion rates [62]. The application of the GoldenGate SNP assay in non model species resulted in slightly lower SNP conversion rates than that obtained for human SNPs. The conversion rate was 91.3% over 1,524 resequenced SNPs in barley [32] and 88.9% over a panel of 450 bovine SNPs [63]. In *Boechera stricta*, a species from the Cruciferae family, a conversion rate of 96.8% was obtained over 96 resequenced SNPs by avoiding highly similar sequences such as for members of complex gene families [64]. For candidate genes that were members of large gene families, the authors searched for markers in flanking genes that were single copy in *Arabidopsis *[64]. Under these circumstances, the design of Illumina probes was efficient and specific. However, such an approach is limited to plant species close to *Arabidopsis*, which genome is completely sequenced.

Two main factors may explain the lower SNP conversion rate obtained with spruces as compared to other species analysed to date with the GoldenGate SNP assay. First, we have adopted the severe criterion of GenTrain scores < 0.25 to reject SNPs, while this factor remains unknown for most of the non human studies submentioned. In the present study, such a conservative criterion translated in SNPs with a high call rate, which was necessary for accurate gene mapping. Second, the complexity of conifer genomes e.g. [37] may obstruct the development of specific probes for the assay. Indeed, our SNP assays incorporated mostly sequences belonging to multigene families including many transcription factors [see Additional file 1]. With the present incomplete knowledge of conifer genomes, it was not possible to take into account gene family structures to improve the oligonucleotide design for the GoldenGate SNP assay. The level of duplication has not been quantified yet in conifer genomes contrary to angiosperm model species such as legumes or grasses. However, phylogenetic analysis of multigene families in conifers has revealed an organization different from that observed in angiosperms. Examples include the *adh *genes [65], *knox-I *[66] and *myb *[67] regulatory genes, with many gene duplications at least recent enough not to be shared with angiosperms. Paralogous SNPs generate a background signal giving rise to cluster compression and therefore, decreasing GenTrain scores and the SNP conversion rate, unless manual editing of the clusters is used to eliminate all SNPs that do no cluster well [29]. If the conifer genome is highly duplicated, as suspected, interpreting GoldenGate SNP data in the context of maximizing the conversion rate could be very challenging and imply much lower call rates per SNP recovered. Without an exhaustive knowledge of gene sequences within a gene family, there is no easy way to avoid such SNPs with potential lack of specificity of flanking sequences. This trend is even more likely, given that all SNPs tested herein were in gene sequences. Indeed, the probability for these SNPs to represent paralogous variation across family members is likely higher than that for SNPs located in non coding DNA.

For SNPs exhibiting monomorphism (about 8% of resequenced SNPs in white spruce and 4% in black spruce), it is likely that one of the two allele-specific primers in the GoldenGate assay defaulted for these SNPs, given that resequencing was performed to discover SNPs and that we are confident that they were not sequencing artifacts. These failures might be related to the same factors as above. Thus, considering the fragmentary knowledge we have of conifer genomes and the emphasis put on sampling SNPs from regulatory genes from large gene families, the overall success rate obtained was decent. The success rate was also repeatable between the two species-specific SNP-arrays that we have independently tested. As the genome of conifers is becoming better known at the sequence level, due to several large-scale EST and BAC sequencing projects, the rates of genotyping success for new SNPs are expected to be even higher in the near future.

### Transcript linkage maps

With around 10,000 to 40,000 Mb [39], spruce and conifer genomes are more than 100 times larger than that of *Arabidopsis *and three times larger than the human genome. For the largely unsequenced conifer genomes, sequencing their coding regions through EST sequencing and gene resequencing currently represents the most efficient approach to comparative and structural genomics using gene linkage maps [19]. The SNP genotyping assays developed in this study enabled to map hundreds of expressed genes in the conifer *Picea*, which represent a large increase over any previous gene mapping effort in gymnosperms e.g. [19,42,41,68-71].

A total of 12 major linkage groups were delineated in each species composite map, which is in agreement with cytogenetic studies indicating similar karyotypes and same numbers of chromosomes (2n = 2x = 24) for both white spruce and black spruce and most other Pinaceae [44]. Because of the agreement between number of linkage groups and number of chromosomes, the present maps could be considered as saturated [Liu 1998]. High genome coverage values also point to this observation. However, a direct pairing between linkage groups and chromosomes was not possible, as *Picea *and most conifer chromosomes are difficult to differentiate based on standard cytological techniques [44]. With the addition of several hundreds new gene markers, the observed total length of individual parental maps was increased by 15% to 25%, and the genome coverage values were increased by nearly 10% compared to previous maps mostly based on anonymous markers [41,42]. With more than 300 gene anchor markers positioned on each parental map, marker density was more than 50% higher and gene density was an order of magnitude higher than previously obtained [41,42]. As compared to AFLP and other types of dominant markers, these codominant SNP gene markers mapped at such high density will contribute towards improving QTL mapping precision and power [72].

More than 200 gene loci were shared between the two composite maps developed herein, most of which being derived from the new gene SNP developing effort. While previous efforts to increase the number of gene or SSR anchor markers relied on using several crosses per species e.g. [41,42,73,74], the ease of mapping large numbers of transcripts using SNPs relaxes the need to implicate more than one cross for increasing the number of mapped anchor markers. However, using an additional cross may be highly useful to validate orthology of gene loci between species when breaks of synteny are observed (see below).

### Interspecific comparisons

Comparisons between the composite maps of white spruce and black spruce revealed high synteny and colinearity between their 12 homoeologous linkage groups, in spite of the divergence of their lineages more than 10 Myr ago [45]. While previous reports of genome comparisons between the two species reached similar conclusions about synteny and colinearity [41], the present observations are based on a more than four-fold increase in the number of mapped anchor loci in common between the two species.

Synteny between genomic regions can only be established if markers are true orthologs [41,42,75]. The breakdown in synteny previously noted between white spruce and black spruce for LG III and LG IV [41] was confirmed in the present study. In addition, three new cases of putative inter-chromosomal translocations between both species were observed, but turned out to be false positives, after checks for locus orthology using haploid megagametophyte DNA sequences or validating gene marker positions in a second cross of white spruce. Thus, these three gene SNPs corresponded to paralogous loci between white spruce and black spruce. Such false positives are likely to be frequent in conifer mapping studies, as previously observed [41].

While synteny was well conserved, exceptions to colinearity between the two genomes were observed. Small inversions between the two species composite maps involving a few closely spaced anchor loci were noted for all linkage groups, but no translocation within linkage groups was observed. Most likely, these inversions resulted from analytical artifacts since the same discrepancies were observed between individual linkage maps within species. Thus, it is safe to assume that these inversions resulted from the integration of both parental datasets for each species rather than from true chromosomal inversions. Such a trend has also been observed when integrating individual linkage maps in other species [5,6,42,73,76].

## Conclusion

The present report illustrates how new highly multiplexed SNP genotyping approaches can be used to accelerate the structural analyses of complex and largely unknown genomes. The present maps are the most advanced genetic maps for spruce with regard to gene density and will open up several opportunities. These maps are currently used for QTL and eQTL detection and their next more densified versions will help target specific regions of the genome for future BAC sequencing [19]. The present maps will also be used to explore in more detail the distribution of multigene families over linkage groups and hence, the organization of the spruce genome and its evolution. Such an approach, though at a small scale, has been previously used for the *knox I *multigene family, underlining a major chromosomal duplication in the spruce genome [66].

Maps with increasing gene densities are also required across the conifers to extend comparative genome studies and decipher the evolution of genome structure. Such comparisons have already been undertaken based on a limited number of anchor markers e.g. [19,41]. However, comparative genome studies in these species remain challenging since SNPs may have to be identified *de novo *in each species. This trend is best exemplified by the small number of gene SNPs shared between mapping populations belonging to different congeneric taxa: only 45 SNPs were shared between white spruce and black spruce mapping populations out of 2,000 candidate gene SNPs discovered in both species. Using more crosses per species could contribute towards increasing the number of shared SNPs between species, but it is unlikely to change the order of magnitude of this number. On a more optimistic note, the primer transfer rate between white spruce and black spruce was high, which might facilitate resequencing efforts towards gene SNP discovery in other spruce species. However, as species from different conifer genera are targeted, the primer transfer rate is expected to diminish [55,19].

Identifying sets of homologous transcripts in other Pinaceae has improved in the recent years through efficient mining of sequence databases e.g. [66,67] and because of large sets of EST sequences for several species [22-25,40]. Nevertheless, when changes in chromosomal structure are observed between species based on gene linkage maps, gene orthology must be verified. Distinguishing between orthology and paralogy can be precarious when the assessment is only based on homology scores, and without an adequate phylogenetic landscape for each gene family involved [77]. In such conditions, a sensitive analysis at the sequence level is warranted before declaring such structural changes [41,42]. Thus, comparative mapping studies between conifer species call for further developments of gene maps and gene sequence collections, but also for genomics and bioinformatics tools enabling to assess more efficiently orthology relationships.

## Authors' contributions

NP, JB: genotyping technology evaluation; BP, IG, NI: genetic mapping analyses; SBe, SBl, FG: resequencing, SNP discovery, design of the SNP arrays, and SNP data analyses; NP, ML, candidate gene identification; NP, BP, JB: manuscript preparation; JB, NI: project design, funding, and overall supervision. All authors have read and approved the final manuscript.

## Supplementary Material

Additional file 1 Data about the SNPs. This table provide references about the genes analysed: unigeneID in ForestTreeDB, annotations, marker names, gene names, links to annotation page in ForestTreeDB, primer sequences used to amplify sequences encompassing the unigenes. Click here for file
